# Isoflurane Induces Endothelial Apoptosis of the Post-Hypoxic Blood-Brain Barrier in a Transdifferentiated Human Umbilical Vein Edothelial Cell Model

**DOI:** 10.1371/journal.pone.0038260

**Published:** 2012-06-18

**Authors:** Michael S. Dittmar, Walter Petermichl, Felix Schlachetzki, Bernhard M. Graf, Michael Gruber

**Affiliations:** 1 Department of Anesthesiology, Regensburg University Medical Center, Regensburg, Germany; 2 Department of Neurology, University of Regensburg, Bezirksklinikum Regensburg, Regensburg, Germany; Julius-Maximilians-Universität Würzburg, Germany

## Abstract

Isoflurane is a popular volatile anesthetic agent used in humans as well as in experimental animal research. In previous animal studies of the blood-brain barrier (BBB), observations towards an increased permeability after exposure to isoflurane are reported. In this study we investigated the effect of a 2-hour isoflurane exposure on apoptosis of the cerebral endothelium following 24 hours of hypoxia in an in vitro BBB model using astrocyte-conditioned human umbilical vein endothelial cells (AC-HUVECs). Apoptosis of AC-HUVECs was investigated using light microscopy of the native culture for morphological changes, Western blot (WB) analysis of Bax and Bcl-2, and a TUNEL assay. Treatment of AC-HUVECs with isoflurane resulted in severe cellular morphological changes and a significant dose-dependent increase in DNA fragmentation, which was observed during the TUNEL assay analysis. WB analysis confirmed increases in pro-apoptotic Bax levels at 4 hours and 24 hours and decreases in anti-apoptotic Bcl-2 in a dose-dependent manner compared with the control group. These negative effects of isoflurane on the BBB after a hypoxic challenge need to be taken into account not only in experimental stroke research, but possibly also in clinical practice.

## Introduction

Isoflurane is a widely used hypnotic volatile anesthetic agent used in both the clinic and experimental animal research. In human stroke, however, anesthesia is not desired in order to retain the patient’s conscience and cooperation for neurological follow-up while in experimental stroke research anesthetic regime are the rule to induce cerebral ischemia by i.e. middle cerebral artery occlusion. In the last decade the concept of the neurovascular unit (NVU) as an integrative system of endothelial cells, astrocytes, neurons, microglia, pericytes and their respective functions has emerged, challenging the classical neurocentric concept of brain ischemia [1], [2], [3]. In addition, neuroprotective drugs appear to have differing, that is deleterious or beneficial effects, depending on the time of administration within the transition from injury to repair at the NVU [4]. Longitudinal stroke studies using non-invasive imaging are particularly suited to assess time specific effects of drug therapy, and enable comparison to the human situation – especially magnetic resonance imaging (MRI) with imaging sequences similar or close to the human brain [5], [6]. However, surveillance of experimental stroke requires repetitive anesthesia for MRI, and this effect on stroke evaluation and outcome as well as interaction to the drug tested is ill characterized but of great importance. Isoflurane has been linked to a variety of effects on endothelial cells, which in the brain represent an essential part of the blood-brain barrier (BBB). Amongst those effects are BBB leakage for macromolecules such as albumin and vasodilatation – effects that ultimately influence stroke outcome [7], [8], [9], [10], [11].

In a recently published stroke study characterizing the biphasic BBB opening following ischemia and reperfusion using serial MRI including T2-relaxometry and post-contrast T1-sequences to assess BBB permeability, we observed progressive cerebral contrast enhancement in the ischemic and non-ischemic brain [12]. This finding is suggestive of para-endothelial contrast agent extravasation through a defective BBB tight-junction complex. An additional rodent study by Hu and coworkers raises further concerns about harmful effects of isoflurane applied subsequent to focal brain ischemia and reperfusion [13].

In the present study using an in vitro BBB model, astrocyte-conditioned human umbilical vein endothelial cells (AC-HUVECs) were subjected to increasing doses of isoflurane both under normoxic conditions and subsequent to sustained hypoxia. We demonstrate that isoflurane induces apoptosis and that this effect is potentiated by hypoxia. These findings are highly relevant to the choice of anesthesia in experimental research and potentially also in the clinical setting.

## Materials and Methods

### In Vitro Model of the BBB

A primary cell culture of HUVECs harvested from donor umbilical cords was stored in liquid nitrogen at −197°C, as previously published [14], [15]. The cells were plated on gelatin-coated tissue cell culture flasks and grown to the first confluence in an atmosphere of 5% CO_2_/95% air at 37°C. The cell culture medium consisted of endothelial cell growth medium (ECGM, Provitro, Berlin, Germany) supplemented with 0.02 ng/L endothelial cell growth factor (Provitro), 5% fetal cattle serum (Sigma Aldrich, Munich, Germany), and 50 mg/L gentamicin (PPA, Cölbe, Germany). Experiments were performed using HUVECs up to passage 5 to minimize the loss of endothelial properties that occurs during multiple passaging.

To induce transdifferentiation of HUVECs into cerebral endothelium–like cells with numerous tight junctions, the cells were grown in 50% (vol/vol) ECGM and 50% astrocytic conditioned medium (ACM), as previously published [16]. In brief, the ACM was prepared by culturing cells from the U-87 line (ATCC, Wesel, Germany), an astrocytic glioblastoma Grade III cell line, in Dulbecco’s modified Eagle medium, low glucose (1 g/L) (Sigma Aldrich), supplemented with 10% fetal cattle serum (Sigma Aldrich), 5 ml 100× modified Eagle medium vitamins (PPA), 5 ml nonessential amino acids (PPA), and 5 mg gentamicin (PPA). The U-87 cells were incubated for 2 days. Following aseptic aspiration, the ACM was frozen at −80°C until use. Verification of the transdifferentiation of HUVECs into cerebral endothelium–like cells was made by measuring the increase in transendothelial electrical resistance (TEER). For this purpose, HUVECs were seeded onto semipermeable 0.2-µm porous filter inserts in 6-well plates (Corning, Kaiserslautern, Germany). After determination of the baseline resistance of the filter, a culture medium composed of 50% ECGM/50% ACM was added above and below the filter element. At intervals of 24 h for a period of 7 days, TEER measurements were made across the monolayer (Millicell ERS, Millipore, Billerica, MA), and the baseline resistance of individual filters was subtracted. The TEER results were converted into specific resistance values by multiplication with the filter area.

### Hypoxia

**Figure 1:**
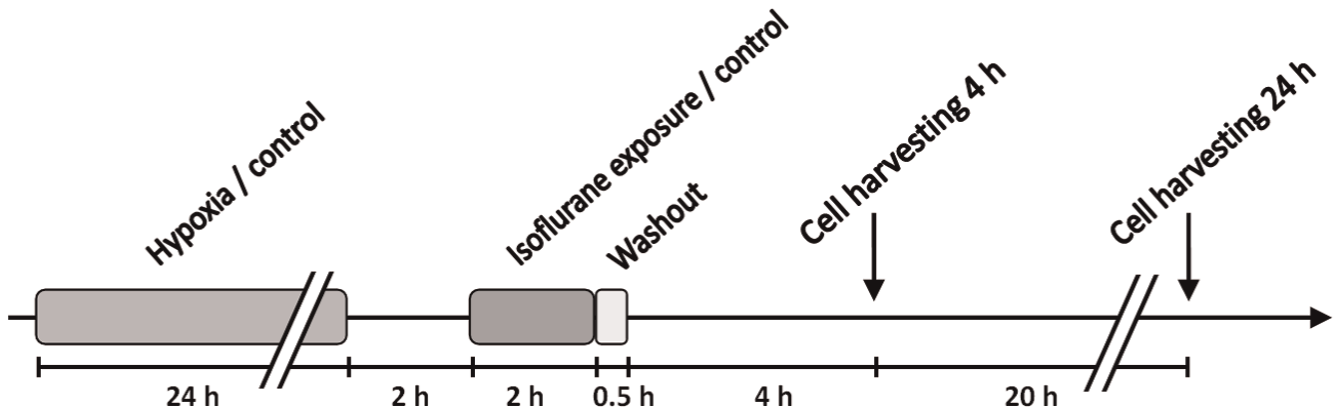
Study Protocol. Diagram of the study setup displaying the time course of experimental interventions performed on transdifferentiated AC-HUVEC.

For oxygen deprivation, transdifferentiated HUVECs were grown to confluence in cell culture flasks with filterless caps, and placed in a BBD 6220 humidified hypoxia chamber (Thermo Scientific Heraeus, Langenselbold, Germany) at 3 vol% O_2_ and 5 vol% CO_2_, with the cap unsealed. After 1 h of equilibration, the caps were sealed completely and the tissue flasks were maintained in the hypoxic atmosphere for an additional 23 h within the chamber. After 24 h of hypoxia, the caps were opened, and the flasks were returned to the normoxic incubator to allow reoxygenation. Following a period of 2 h, the AC-HUVECs were subjected to isoflurane or control treatment (Figure 1).

### Anesthesia Treatment

The AC-HUVECs were treated with the volatile anesthetic agent isoflurane or control conditions by using a modified anesthesia unit Trajan 808 (Draeger, Lübeck, Germany), which delivered compressed air (95%)/CO_2_ (5%). An anesthesia gas vapor for the evaporation of isoflurane (Forane, Abbott India, Verna Salcette, India) was installed. The gas mixture was conducted into cell culture flasks, and the temperature of the flasks was maintained at 37°C by placing them on feedback-regulated heated gel pads. The gas composition in the culture flasks was continuously monitored using a Capnomak Ultima monitor (Datex Engstrom, Fairfield, CT, United States). The AC-HUVECs were treated according to a study protocol with isoflurane concentrations of 0 (control), 0.5, 1, or 2 minimal alveolar concentrations (MAC) for 2 h. One MAC was considered 1.3 vol%. After the treatment period, the anesthetic agent was washed out with 95% air/5% CO_2_ for 30 min. Control experiments were performed in the same manner, but without adding isoflurane to the gas mixture. Afterwards, the culture flasks were returned to the incubator for a recovery period of either 4 or 24 h before harvesting and analysis (see Figure 1 for a diagram of the study design).

### TUNEL Staining and Microscopy

To estimate the degree of apoptosis, we used the terminal deoxynucleotidyl (TdT)–mediated dUTP-biotin nick end labeling (TUNEL) technique (n = 2 independent experiments per group). For this, AC-HUVECs were grown on cover glasses until confluence. After they had been exposed to isoflurane or control conditions and allowed the recovery period, the cells were fixed with 4% paraformalin solution. Following permeabilization of the samples with Triton X100 0.1% (100 µl per cover slip), staining was performed using an In-situ Cell Death Detection Kit (Fluorescein 116847959-10 kit, Roche, Mannheim, Germany). The read-out was performed using a fluorescence microscope (Leica DM RBC, Leica Microsystems, Wetzlar, Germany) equipped with an EBQ 100 fluorescence detector (Leica Microsystems) at an emission wavelength of 565 nm; absolute fluorescence intensities were recorded by the FM Visi View software (V 2.0.3, Leica Microsystems) under identical microscopic settings (28 ms exposure time, 40 times magnification). Per slide, three pictures allocated in a pre-defined manner were taken, and the mean of overall image fluorescence was used for further calculations. As positive controls, we used AC-HUVECs that had been permeabilized with Triton X100 (PAA) for 10 min at room temperature and treated with 3000 U/ml recombinant DNAase 1 (Sigma Aldrich) before TUNEL staining.

### Western Blot Analysis

Western blot (WB) analysis was performed to investigate expression of the apoptosis marker Bcl-2–associated X protein (Bax) and the anti-apoptotic B-cell lymphoma protein 2 (Bcl-2) in AC-HUVECs (n = 3 independent experiments per group) [17], [18]. The cells were trypsinized (4 ml per 75 cm^3^ flask 2.5×Trypsin EDTA w/o Ca^++^/Mg^++^, Sigma Aldrich) and harvested, and the cell pellet was lysed on ice using 100 µl RIPA buffer (5 ml Triton X100 [PAA], EDTA 190 mg [Sigma Aldrich], 0.5 mg SDS [Sigma Aldrich], and 2.5 g sodium deoxycholate in 500 ml PBS w/o Ca^++^/Mg^++^ [PAA]) containing protease inhibitors (cOmplete ULTRA, Roche) and phosphatase inhibitors (PhosSTOP, Roche) at a concentration of 1 tablet/ml, respectively. After 30 min of centrifugation at 8,400 *g* and 4°C, the protein-rich supernatant was removed and stored at −80°C until analysis.

For the gel electrophoresis, 10% acrylamide SDS separating gels (Sigma Aldrich) were prepared. Protein samples were diluted 3∶1 with 4× Laemmli buffer. After instilling the electrophoresis buffer TGS (BioRAD, Munich, Germany), 40 µg of protein per lane were loaded. Quantification of total protein content was performed using a BCA test kit (BCA Assay Kit, Thermo Fisher Scientific GmbH, Ulm, Germany). As a size standard, fluorescent Page Ruler Prestained Protein Ladder (Fermentas GmbH, St. Leon-Rot, Germany) was applied. As positive controls, we used RAW 264.7 (IP) Cell Lysate (SC- 2211, Santa Cruz Biotechnology, Heidelberg, Germany) for Bax and WEHI 231 Cell Lysate (SC- 2213, Santa Cruz) for Bcl-2. After electrophoresis the proteins were blotted for 60 min at 300 mA on Membrane Hybond-CExtra nitrocellulose (Amersham, Bucks, UK). Thereafter the blots were rinsed three times with 1× washing buffer TBS (Sigma Aldrich) with Tween 20 (PAA) and blocked for 60 min with milk powder at room temperature. The primary antibody anti-ß-actin, produced in mouse (A5316, Sigma Aldrich) and diluted 1∶5,000 in blocking buffer (LI-COR Biosciences GmbH, Bad Homburg, Germany), and the primary antibody anti-Bax or anti-Bcl-2, produced in rabbit (Cell Signaling, Frankfurt am Main, Germany) and diluted 1∶1000 in LI-COR blocking buffer, were applied. Blots were incubated for 16 h at 4°C. After rinsing, the fluorescence-labeled secondary antibodies anti-mouse and anti-rabbit, produced in donkey (700/800 IRDye, LI-COR) and diluted 1∶15,000 in washing buffer (BioRAD), were added. For 1 h the blots were incubated in darkness at room temperature. The WBs were read using a blot reader (Odyssey, LI-COR). Bax and Bcl-2 levels were normalized for ß-actin.

### Statistical Analysis

Statistical analysis was performed using PASW Statistics 18 (SPSS Inc., Chicago, IL, US). To compare protein marker levels, we used the Kruskal-Wallis test; this was followed by the Mann-Whitney test to compare individual groups and time points. To confirm a concentration-response relation, we performed a curve fit regression analysis to test linear, quadratic, and exponential dependencies (independent variable: isoflurane concentration; dependent variable: TUNEL intensity or Bax/Bcl-2 protein expression, respectively). This explorative approach has been utilized to make sure to detect both linear and complex dependencies. The best fit in terms of R^2^ is reported. For the TUNEL data, only the regression analysis was performed. Differences were considered significant if P≤0.05. Values are expressed as median ± interquartile ranges.

## Results

### TEER Measurements

During growth of the freshly seeded HUVECs, the TEER increased until it reached a maximum on Day 3–4, after which the TEER began to decline gradually. The ACM treatment almost doubled the maximum specific TEER to values slightly above 600 Ωcm^2^ (data not shown). All isoflurane and hypoxia experiments started on Day 4.

### No Hypoxia

#### TUNEL assay and microscopy

**Figure 2:**
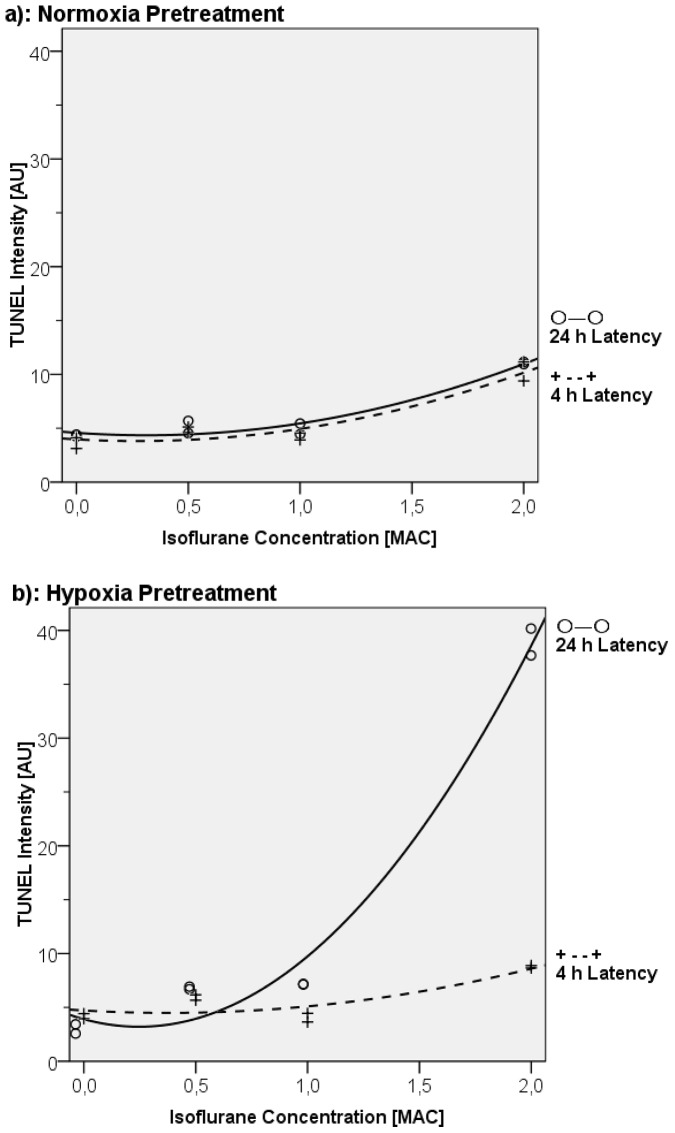
TUNEL Regression Analysis. Quantification and quadratic regression analysis of TUNEL fluorescence in relation to hypoxia, isoflurane concentrations, and delay to analysis subsequent to 2 hours of isoflurane exposure. a) No hypoxia. 4-hour latency period: R^2^ = 0.91, P = 0.003; 24-hour latency period: R^2^ = 0.95, P<0.001. b) Hypoxia for 24 hours followed by 2 hours of reoxygenation prior to isoflurane treatment. 4-hour latency period: R^2^ = 0.75, P = 0.031; 24-hour latency period: R^2^ = 0.98, P<0.001.

Fluorescence levels in the AC-HUVECs, demonstrated by TUNEL staining, exhibited a dose dependency, up to a 2.5-fold increase, which was best described using a quadratic regression model, both after 4 h (R^2^ = 0.91, P = 0.003) and 24 h (R^2^ = 0.95, P<0.001)(Figure 2A). Microscopic analysis performed 4 h after isoflurane exposure still showed a confluent layer of visually normal cell bodies with normal nuclei, similar to those seen in the untreated control group. After 24 h following isoflurane treatment, gaps occurred in the cell layer along with a stronger condensation of nuclei, and there was budding at cell boundaries, irrespective of which isoflurane concentration had been applied (data not shown).

#### Bax and Bcl-2

**Figure 3:**
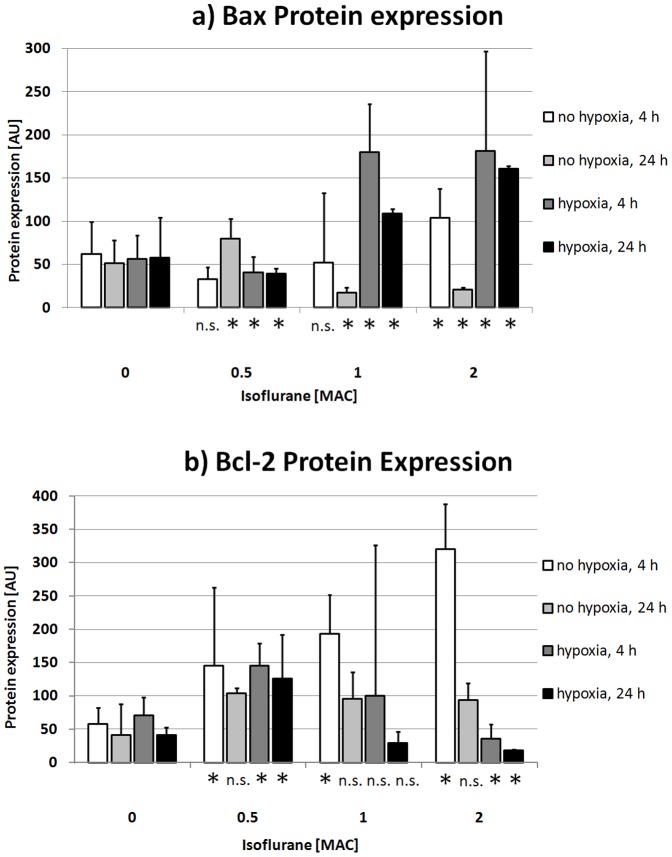
Bax/Bcl-2 Western Blot bar graphs. Median value and interquartile range of Bax (a) and Bcl-2 (b) protein expression in relation to hypoxic challenge and recovery period (4/24 h). N.s.: not significant, *: statistically significant difference to control/0 MAC in Mann-Whitney test.

**Figure 4:**
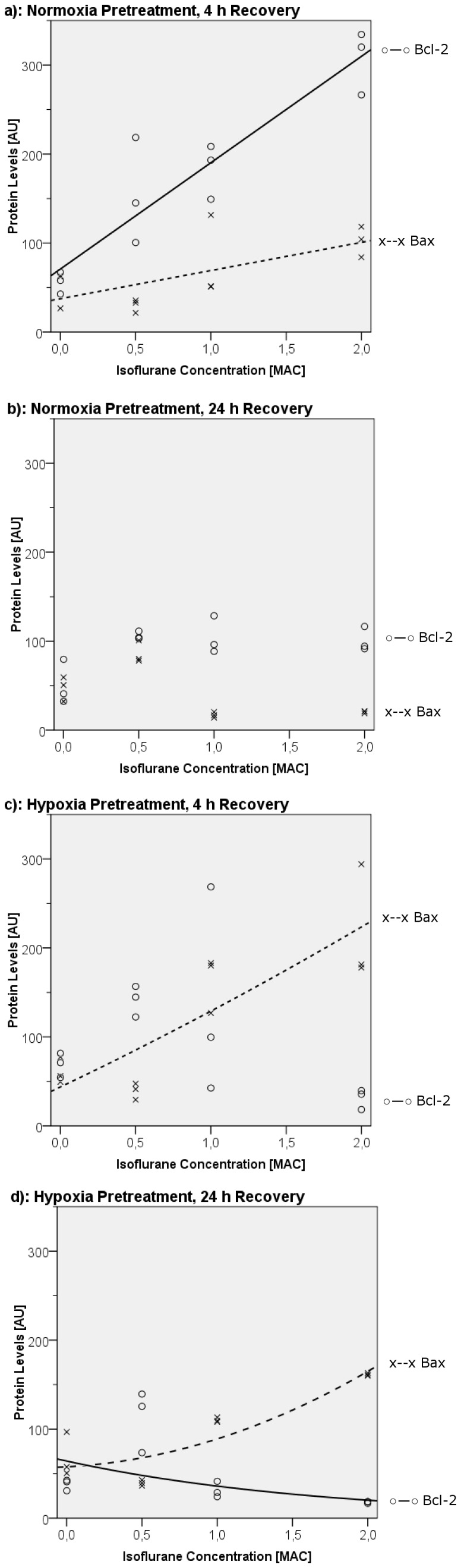
Bax/Bcl-2 Western Blot Curve Fit Analysis. Western blot data and curve fit of Bax (drawn as crosses and dashed lines) and Bcl-2 (rings, solid lines) in relation to hypoxia, isoflurane concentration, and delay to analysis subsequent to 2 hours of isoflurane exposure. a) No hypoxia. 4-hour latency period: significant positive linear isoflurane concentration dependency for both Bax (R^2^ = 0.44, P = 0.02) and Bcl-2 (R^2^ = 0.87, P<0.001). b) No hypoxia, 24-hour latency period: no significant concentration dependency. c & d) Hypoxia for 24 hours followed by 2 hours of reoxygenation prior to isoflurane treatment. c) 4-hour latency period: positive quadratic dose dependency for Bax (R^2^ = 0.71, P = 0.004); d) 24-hour latency period: positive quadratic dose dependency for Bax (R^2^ = 0.80, P = 0.001) and negative exponential dependency for Bcl-2 (R^2^ = 0.39, P = 0.03).

In experiments not involving hypoxic challenge, isoflurane exposure up to 1 MAC did not lead to significant changes in Bax protein levels in the overall comparison at 4 h posttreatment compared to baseline (P = 0.08, Kruskal-Wallis test). However, at 2 MAC we did observe a significant increase in the Bax level over baseline (104±34 arbitrary units (AU) vs. 62±37 AU, P = 0.05, Mann-Whitney test; Figure 3A). Thus, a weak linear dose-response relationship was present (R^2^ = 0.44, P = 0.02, Figure 4A). In contrast, after 24 h there were significant changes in Bax levels compared to baseline (P = 0.02), with an increase after treatment with 0.5 MAC isoflurane (80±23 AU vs. 51±27 AU, P = 0.05) and a decrease in Bax levels in the 1-MAC and 2-MAC groups to 17±6 AU and 21±2 AU, respectively (control: 51±27 AU, P = 0.05, Figure 3A). There was no linear or quadratic dependency in the regression analysis (Figure 4B).

The anti-apoptotic marker Bcl-2 was observed to have an early increase in protein levels from 58±24 AU (control) to 145±118 AU, 193±59 AU, and 320±68 AU in response to rising isoflurane concentrations (P = 0.02 in the global Kruskal-Wallis analysis, and P = 0.05 for each isoflurane concentration, Mann-Whitney test, Figure 3B); the increase was concentration dependent (linear regression: R^2^ = 0.87, P<0.001; Figure 4A). The significant difference, compared to control cells, was lost at the 24-hour time point (P = 0.09, Kruskal-Wallis test) as was the concentration dependency (R^2^ = 0.27, P = 0.82; Figure 4B).

### Hypoxia

#### TUNNEL assay and microscopy

**Figure 5:**
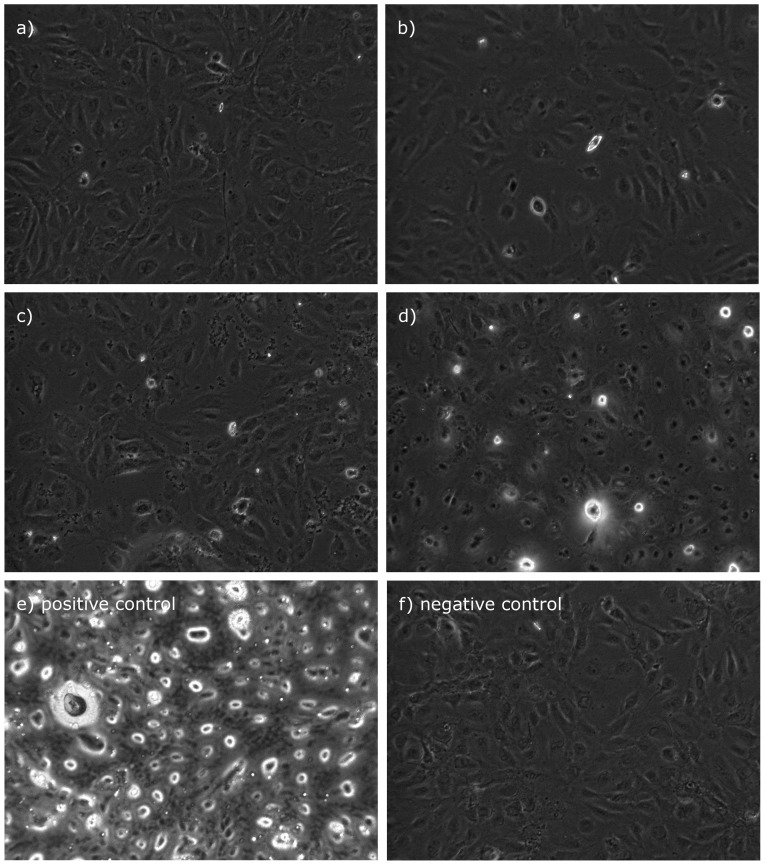
TUNEL Fluorescence Microscopy. Representative light microscopy images of TUNEL fluorescence in labeled AC-HUVECs after 24 hours of hypoxia, 2 hours of isoflurane exposure with 0 MAC (control; fig. 4a), 0.5 MAC (b), 1 MAC (c), 2 MAC (d), and a subsequent 24-hour recovery. In addition, positive (e) and negative (f) controls are presented.

TUNEL intensity increased almost 2-fold at high isoflurane concentrations (2 MAC) 4 h posttreatment and increased further to as much as 8 times baseline at 24 h. A quadratic equation fit the data best (R^2^ = 0.75, P = 0.031 at 4 h, and R^2^ = 0.98, P<0.001 at 24 h, Figure 2B). Microscopic examination of AC-HUVECs exposed to normoxic conditions (controls) revealed normal cell shapes, morphological characteristics, and confluence. Similarly, after 4 h of recovery from isoflurane exposure and hypoxia the cells appeared unremarkable. In contrast, 24 h after exposure to isoflurane and hypoxia we could detect multiple interruptions of the cell layer, condensations of nuclei, and budding at cell boundaries in the cell layer. These effects were greatest in the 2-MAC isoflurane group, compared with groups receiving lesser concentrations. In addition, TUNEL fluorescence increased with rising isoflurane concentrations (Figure 5).

#### Bax and Bcl-2

In AC-HUVECs that had been rendered hypoxic before isoflurane treatment, Bax expression differed significantly from that of isoflurane-naïve control cells, both after 4 and 24 h of latency (P = 0.02, Kruskal-Wallis test respectively). There were increases in Bax levels in the early group, from 56±28 AU (control conditions) to 180±56 AU (1 MAC) and 181±116 AU (2 MAC), with an intermediate decrease in the Bax level at 0.5 MAC (41±18 AU; P = 0.05 for each group, Figure 3A). In the curve fit analysis, the correlation could best be described by a quadratic model (R^2^ = 0.71, P = 0.004, Figure 4C). At 24 h after isoflurane exposure we found a similar expression pattern: subsequent to a decrease in the Bax level at a low dose (0.5 MAC) of isoflurane (39±6 AU vs. 58±46 AU for control), increases in Bax levels were detected for higher isoflurane concentrations (109±5 AU at 1 MAC and 161±3 AU at 2 MAC, P = 0.05 respectively, Figure 3A). Again, a quadratic regression was the best fit (R^2^ = 0.80, P = 0.001, Figure 4D).

Compared to Bax expression, Bcl-2 expression behaved in an almost inverse manner: After both 4 and 24 h, increases in Bcl-2 levels were observed in cells treated with 0.5 MAC compared to control cells (145±34 AU vs. 71±27 AU after 4 h; 126±66 AU vs. 41±12 AU after 24 h; P = 0.05, Mann-Whitney test). With increasing concentrations of isoflurane, however, Bcl-2 levels decreased (at 4 h: 100±226 AU at 1 MAC [n.s.] and 36±21 AU at 2 MAC [P = 0.05] vs. control, Mann-Whitney test, Figure 4C; after 24 h: 29±17 AU at 1 MAC [n.s.] and 18±2 AU at 2 MAC, P = 0.05, Figure 3B). For both time points the global statistical comparison confirmed the differences (P = 0.04 and 0.02, respectively, Kruskal-Wallis test). In the curve fit analysis, only at 24 h was a weak exponential dose-response relationship detectable (R^2^ = 0.39, P = 0.03, Figure 4D).

## Discussion

In this study we investigated the influence of the volatile anesthetic agent isoflurane on AC-HUVECs, a well-established in vitro model used in BBB studies. We could show a delayed and dose-dependent increase in the pro-apoptic marker Bax and a decrease in the anti-apoptotic marker Bcl-2. Our observations were further supported by TUNEL staining and by morphological assessment, confirming the presence of increased apoptosis. This finding is important for clinical anesthesiology and also for experimental research, especially in investigations of CNS diseases such as stroke.

For many years, stroke research focused on neuronal pathology and neuroprotection, yet did not result in clinically beneficial neuroprotective regimens. Recently, a more holistic approach has been introduced, shifting interest from neurons toward the neurovascular unit (NVU) [1]. In the NVU concept, interaction between neurons, astrocytes, microglia, pericytes and endothelium, the latter forming the main component of the BBB, occur, ultimately affecting neurological outcome in this primarily vascular disorder. One of the most important pathophysiological consequences of cerebral ischemia is the loss of barrier function of the BBB (often referred to as disruption or leakage). This effect comprises not only loss of tight junction function but also degradation of basal lamina matrix proteins and alterations of the *glia limitans* formed by astrocyte end feet [19]. BBB leakage following cerebral ischemia in humans can be observed from 3 h to 7 days [20] and, on the endothelial level, is dependent on the destabilization of the tight junction complex and consecutively the para-endothelial influx of low-molecular-weight contrast agents up to large molecules such as albumin [21].

In experimental stroke research, BBB disruption has shown to be a highly dynamic process, as first described by Olsson et al. in 1971 [22], and at least two distinct BBB openings to contrast agents such as Evans blue and [3 H] sucrose have been demonstrated [22], [23], [24]. The molecular basis of increased BBB permeability comprises alterations in the expression of tight-junction related proteins and conforming changes of tight junction proteins within the cell membranes, especially occludin, claudin-2 and claudin-5. This can be achieved via a large variety of pathways. Chemokines, excitatory amino acids, and matrix metalloproteinases (MMP) are only some important candidates induced by ischemia and released by pericytes, astroglia and inflammatory cells [19], [25]. In rats it has been demonstrated that acute astrocyte loss can induce reversible loss of the normal para-cellular localization of the transmembrane proteins occludin and claudin-5 and cytoplasmic ZO-1, which correlated with increased para-endothelial efflux due to defective tight junctions, changes in transendothelial flux and loss of structural support due to basal membrane and extracellular matrix degradation [25], [26], [27].

The concept of biphasic opening of the BBB has been demonstrated after focal ischemia/reperfusion, with early short-lasting para-endothelial leakage within the first 4 to 6 h, partial closure at 24 h, and a delayed long-lasting opening that begins with a peak at 48 h [12]. The latter may last up to 1 month, depending on the length and severity of the ischemia [28]. Abrogation of BBB leakage by blocking MMPs in the early phase has proved to be beneficial, whereas delayed treatment worsens outcome [5], [29]. Thus, the timing of an intervention needs to be included as a parameter in the scope of stroke investigations to a much greater extent than it was in the past.

Numerous studies on the volatile anesthetic agent isoflurane and its effect on the pathophysiology of focal cerebral ischemia when administered during the ischemic period have produced conflicting results [30], [31], [32], [33], [34], [35], [36], [37], [38], [39]. However, there is a lack of sufficient data on the influence of isoflurane when administration is delayed until the reperfusion period. In the early post-ischemic phase the volatile anesthetic might exert a neuroprotective effect, referred to as postconditioning, by activating the sphingosine-1-phosphate/phosphatidylinositol-3-kinase/Akt pathway [40] or by interfering with mitochondrial adenosine 5′-triphosphate–sensitive potassium channels [41]. Tertrault et al., on the other hand, published a paper in which isoflurane treatment of healthy cat brains resulted in extravasation of Evans blue [7]. In a rat model of transient middle cerebral artery occlusion, isoflurane worsens not only infarct volume, but also brain edema and hemorrhagic transformation if applied for one hour immediately after reperfusion [13], which supports the notion of isoflurane mediated cerebro-vascular injury.

Isoflurane is known to promote the neuronal expression of Bcl-2, which is a supposed mechanism of its neuroprotective effect [38], [39]. In contrast, in this study we were able to demonstrate that exposure to isoflurane in clinically relevant concentrations leads to a dose-dependent shift in endothelial Bax and Bcl-2 protein expression, followed by an increase in delayed apoptosis in AC-HUVECs. These changes were potentiated by hypoxia prior to isoflurane treatment. The expression of the pro-apoptotic Bax and the anti-apoptotic Bcl-2 behaved in a complex pattern. Bcl-2 showed a dose-dependent, timely limited increase in expression under normoxic conditions (Figures 3B, 4A, and 4B), but a delayed down regulation with increasing isoflurane concentrations in posthypoxic cells (Figures 3B, 4D). Its counterpart Bax, on the other hand, was only slightly influenced by exposure to the anesthetic agent as long as the endothelium was not rendered hypoxic. After hypoxia, Bax levels rose substantially in parallel with isoflurane dosing. Thus, in posthypoxic AC-HUVECs in which there was a 24-hour latency period subsequent to isoflurane treatment (Figures 3, 4D), Bax and Bcl-2 displayed inverse behaviors, favoring the development of apoptosis, which, in turn, manifested in DNA degradation at the end stage of apoptosis, as demonstrated using TUNEL staining and morphological observations.

These findings have several implications. Neurons and endothelial cells could be affected by postischemic isoflurane exposure in an opposite fashion. Although neurons seem to be protected by isoflurane [38], [39], [40], [41], cerebral endothelial cells forming the BBB may be severely injured. This stresses the importance of a taking a holistic view on the effects of peri-ischemic interventions to the brain. Secondly, the role of isoflurane in CNS research has to be re-evaluated: if isoflurane proves to have a profound influence on BBB integrity, its usefulness in stroke research must be questioned. Last, but not least, isoflurane is a popular anesthetic agent in patient care. Despite the vast clinical usage and well-established safety profile of the drug, there may be certain circumstances that we are currently not aware of in which isoflurane should be replaced. If the results of our study hold true in further experimental approaches and also in the clinical setting, it may be best if patients at risk for cerebral edema, like after brain trauma or during carotid desobliteration, receive an alternative hypnotic compound to perform general anesthesia.

### Study Limitations

This study was performed on an in vitro model of the BBB, in which human umbilical cells were transdifferentiated to a cerebro-vascular phenotype by astrocyte conditioned culture medium, and kept without contact to their natural cellular environment (neurons, pericytes, blood etc.). Thus, their behavior not necessarily reflects the in vivo situation. Since the analysis was restricted to two distinct time points, important phenomena which manifested outside the observational time window might have been missed. In addition, case numbers were low, rendering a certain extent of uncertainty to the statistical analysis and establishing the need for further experimental confirmation.

In summary, isoflurane has a profound influence on endothelial cells within the first day after sustained hypoxia in an in vitro model of the BBB, were it induces endothelial apoptosis in a delayed, dose-dependent manner. Therefore this anesthetic agent should be avoided in in vitro experiments focusing on aspects of BBB morphology or function. Further studies must be conducted to clarify whether the following are the case: i) other volatile anesthetic agents have similar effects on the BBB; ii) timing of anesthesia is relevant (i.e. whether there is a vulnerable interval) after cerebral ischemia; and iii) the isoflurane effect on the BBB is relevant to outcome after cerebral ischemia in vivo.
